# Effect of vibration on orthodontic tooth movement in a double blind prospective randomized controlled trial

**DOI:** 10.1038/s41598-022-05395-5

**Published:** 2022-01-25

**Authors:** Atsushi Mayama, Masahiro Seiryu, Teruko Takano-Yamamoto

**Affiliations:** 1 Division of Orthodontics and Dentofacial Orthopedics, Graduate School of Dentistry, Tohoku University, 4-1, Seiryomachi, Aoba-ku, Sendai, Miyagi 980-8575 Japan; 2 Department of Biomaterials and Bioengineering, Faculty of Dental Medicine, Hokkaido University, Hokkaido, 060-8586 Japan

**Keywords:** Randomized controlled trials, Medical research, Clinical trial design, Translational research

## Abstract

The purpose of the present study was to investigate the effect of vibration on orthodontic tooth movement and safety assessment based on our previous basic research in animal experiments. A double-blind prospective randomized controlled trial using split-mouth design was conducted in patients with malocclusion. The left and right sides of maxillary arch were randomly assigned to vibration (TM + V) and non-vibration (TM) groups. After leveling, vibrations (5.2 ± 0.5 g-forces (gf), 102.2 ± 2.6 Hertz (Hz)) were supplementary applied to the canine retracted with 100 gf in TM + V group for 3 min at the monthly visit under double-blind fashion, and the canine on the other side without vibration was used as TM group. The amount of tooth movement was measured blindly using a constructed three-dimensional dentition model. The amount of canine movement per visit was 0.89 ± 0.55 mm in TM group (n = 23) and 1.21 ± 0.60 mm in TM + V group (n = 23), respectively. There was no significant difference of pain and discomfort, and root resorption between the two groups. This study indicates that static orthodontic force with supplementary vibration significantly accelerated tooth movement in canine retraction and reduced the number of visits without causing side effects.

## Introduction

Active orthodontic treatment with the edgewise appliance takes several years to complete. During treatment, brackets and wires hamper proper oral hygiene, and there are some risks of periodontal disease and dental caries^1^. The prolonged treatment period also causes side effects such as root resorption and prolonged pain due to orthodontic tooth movement, which increases the patient's burden^2^. Therefore, the development of a new method of accelerating orthodontic tooth movement is required to shorten the treatment period.

In recent years, there have been many reports about applying surgical, pharmacological and physical stimulation with conventional orthodontic force to shorten the duration of orthodontic treatment in basic and clinical studies. Surgical approaches such as corticotomy^3^ and piezoelectric ultrasonic bone punctures^4^ stimulate the rate of orthodontic tooth movement. However, corticotomy causes severe pain and discomfort during surgery because of the separation of alveolar mucosa, and removal of cortical bone. Bone puncture also involves invasion and a healing process for the gingiva and bone. Therefore, it is difficult to apply surgical techniques to all orthodontic patients. In human clinical trials as a pharmacological approach, application of prostaglandin ^5^ and relaxin^6^ has been reported. However, this has been reported to promote root resorption^7^, and it is difficult to obtain the patient’s consent due to uncertain pharmacological side effects. Therefore, the pharmacological approach is not suitable for clinical application at present.

Recently, in the field of orthopedic surgery, mechanical stimulation such as Low Intensity Pulsed Ultra-Sound (LIPUS) has promoted fracture healing^8^. Low magnitude, high-frequency vibration (LMHF vibration, LM; less than 1 g, where g = 9.81 m/s^2^, HF; 20–90 Hz), which has been applied as a safe and non-invasive treatment method, has prevented postmenopausal bone loss^9^. LMHF vibration has increased bone and muscle mass in osteoporotic patients^10^ and reduced bone and muscle mass loss in bedridden patients^11^.

For orthodontic treatment, various physical approaches, including low-level laser^12–16^, electromagnetic fields^17^, photobiomodulation^18,19^, and vibration^20–23^, have been reported to accelerate orthodontic tooth movement.

Although tissue damage and side effects caused by low level laser and electromagnetic fields have not been fully clarified, a report of photobiomodulation therapies (Orthopulse™) showed an increased risk of root resorption^26^. Furthermore, there are no reports of a randomized controlled trial in accelerated orthodontic tooth movement using low-level laser, electromagnetic fields, and photobiomodulation. Thus, there is no consensus on the use of such physical approaches as clinical applications for orthodontic treatment.

The vibration device AcceleDent™ has been currently developed to promote orthodontic tooth movement. There are reports showing its effectiveness in dentition leveling during orthodontic treatment^21^ and in canine movement^22^. In contrast, Woodhouse et al.^24^ found no significant difference in mandibular dentition leveling between two groups, with or without AcceleDent™, and DiBiase et al.^25^ also showed AcceleDent™ produced no significant increase in space closing. The vibration characteristics of AcceleDent™ are a force of 0.25 N and a frequency of 30 Hz. However, there has been no evidence of AcceleDent™ being applied to dentition under optimal characteristics of vibration for accelerating tooth movement. In any case, the problem with clinical studies using AcceleDent™ is that it is unclear if it was used accurately and reliably by subjects at home for 20 min/day. So far, evaluation of the clinical efficacy and safety of AcceleDent™ is controversial.

Previously, we have developed a vibration generator for rats, applied various vibrations to the teeth, and evaluated the stimulatory effect of supplementary vibration on experimental tooth movement in rats^27^. The characteristics of vibration (3gf at 70H) and the method of applying vibration to the teeth of rats were established^27^. Furthermore, we suggested that static loading and supplementary vibration promoted bone remodeling via activation of nuclear factor-kappa B to stimulate osteoclastic bone resorption and accelerated orthodontic tooth movement ^27,28^.

The purpose of the present study was to investigate the effect of vibration of 5.2 ± 0.5 gf at 102.2 ± 2.6 Hz similar to vibration given to rat, applied to patients on orthodontic tooth movement and assess its safety. A prospective randomized controlled trial using a split-mouth design was conducted in patients with malocclusion. This vibration was applied to canines in a double-blind fashion, and the amount of tooth movement was measured blindly using a constructed three-dimensional plaster model. For safety assessment, pain and discomfort were evaluated using the visual analog scale (VAS) method, and root resorption was evaluated using radiographic images.

## Results

### Participants

**Figure 1:**
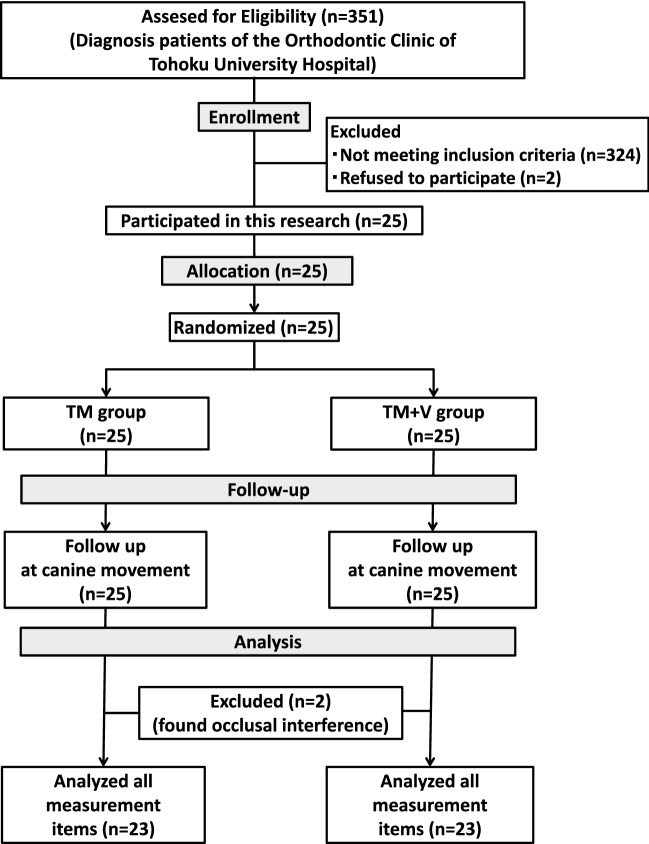
CONSORT diagram. Twenty-seven subjects provided consent explanations, two of whom refused to participate. The left and right side dentition of the twenty-five subjects were randomly classified into the TM group and the TM + V group. Two of the twenty-five subjects had occlusal interference and were excluded from the analysis.

The CONSORT diagram is shown in Fig. 1. The final number of subjects was 25 (2 refused to participate), 4 males and 21 females, average age of 20.2 ± 7.0 years (minimum (min): 13.5, maximum(max): 40.4 years). In terms of malocclusion, the classifications were maxillary protrusion: 4, crowding: 10, maxillary protrusion and crowding: 11; skeletal Class I: 10, Class II: 10, and Class III: 5; and Angle Class I: 11, Class II: 12, Class III: 2 (Table 1). Two subjects with occlusal interference in the maxillary canine and mandibular dentition during canine retraction were excluded from the study. In total, 23 subjects (TM group, n = 23; TM + V group, n = 23) were evaluated (Fig. 1).

**Table 1:** Summary of age, gender, skeletal, and Angle classification of subjects.

Variable	Subject
**Age, years**	
Mean	20.2
Standard deviation	7.0
Maximum	40.4
Minimum	13.5
**Gender, no. (%)**	
Male	4 (16)
Female	21 (84)
**Skeletal classification, no. (%)**	
Class I	10 (40)
Class II	10 (40)
Class III	5 (20)
**Angle classification, no. (%)**	
Angle Cl.I	11 (44)
Angle Cl.II	12 (48)
Angle Cl.III	2 (8)

### Accelerated effect of supplementary vibration on orthodontic tooth movement

The canine movement was measured at each visit up to the 8th visit and the mean period was 6.9 ± 2.9 months (min: 1.6 months, max: 9.9 months, 95% confidence interval (CI): 4.8 to 7.2 months). The numbers of subjects at each visit were 23 at 1st and 2nd visit, 20 at 3rd visit, 14 at 4th, 10 at 5th, 7 at 6th, 6 at 7th and 5 at 8th visit.

**Figure 2:**
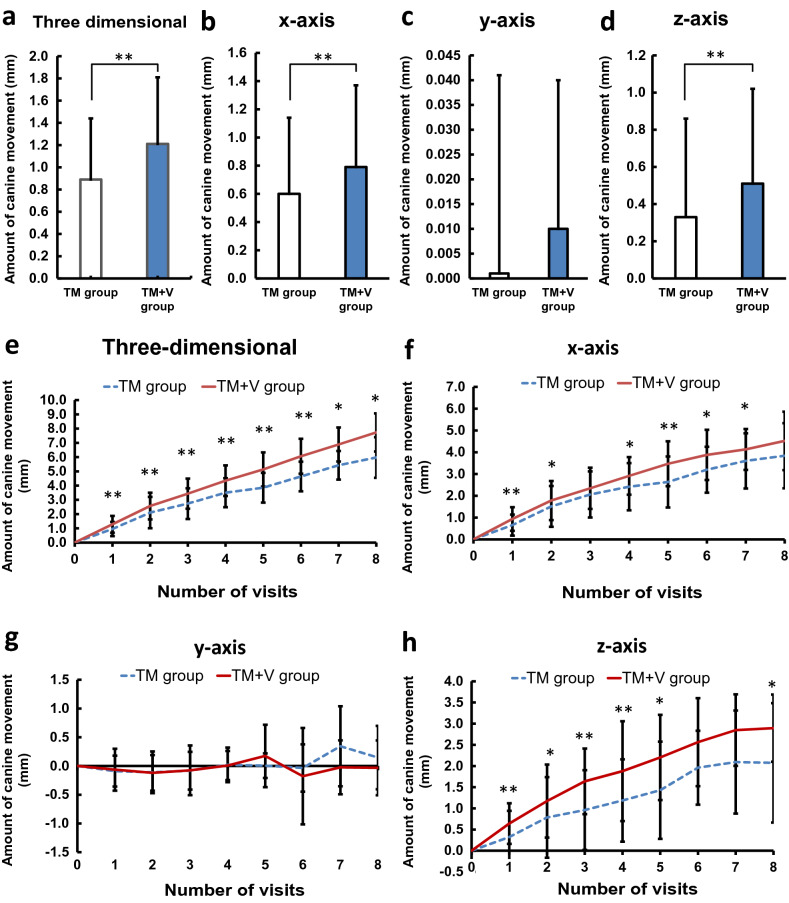
Amount of canine movement. **(a)** Amount of three-dimensional movement of canine. **(b–d)** Amount of canine movement on each coordinate axis. **(b)** Amount of canine movement on x-axis. **(c)** Amount of canine movement on y-axis. **(d)** Amount of canine movement on z-axis. **(e)** Amount of canine movement for each visit. **(f–h)** Amount of canine movement for each visit on coordinate axis. **(f)** Amount of canine movement for each visit on x-axis. **(g)** Amount of canine movement for each visit on y-axis. (h) Amount of canine movement for each visit on z-axis. *P < 0.05, **P < 0.01.

The mean amount of canine movement at each visit was 0.89 ± 0.55 mm in the non-vibration group (TM group) (95% CI, 0.81 to 1.07 mm), and 1.21 ± 0.60 mm in the vibration group (TM + V group) (95% CI, 1.01 to 1.30 mm), respectively. A significant difference was observed between the two groups (**P < 0.01) (Fig. 2a). In the comparison of each coordinate-axis, the mean amount of canine movement per visit in the TM group was 0.60 ± 0.54 mm on the x-axis (95% CI: 0.52 to 0.72 mm), 0.001 ± 0.04 mm on the y-axis (95% CI, -0.06 to 0.07 mm), and 0.33 ± 0.53 on the z-axis (95% CI, 0.24 to 0.43 mm), and those in the TM + V group was 0.79 to 0.58 mm on the x-axis (95% CI: 0.79 to 0.90 mm), 0.01 ± 0.03 mm on the y-axis (95% CI, -0.08 to 0.08 mm), and 0.51 ± 0.51 mm on the z-axis (95% CI, 0.42 to 0.60), and a significant difference was observed on the x-axis and the z-axis (**P < 0.01) (Fig. 2b–d).

In the comparison of three-dimensional movement for each visit, significant differences were observed at all visits (1st, 2nd, 3rd, 4th, 5th and 6th: **P < 0.01, 7th and 8th: *P < 0.05) (Fig. 2e). In the evaluation of each coordinate axis, significant differences were observed except for on the 3rd and 8th visits on the x-axis (1st and 5th: **P < 0.01, 2nd, 4th, 6th and 7th: *P < 0.05), the 6th and 7th visits on the z-axis (1st, 3rd, and 4th: **P < 0.01, 2nd, 5th and 8th: *P < 0.05), and on the y-axis (Fig. 2f–h).

**Figure 3:**
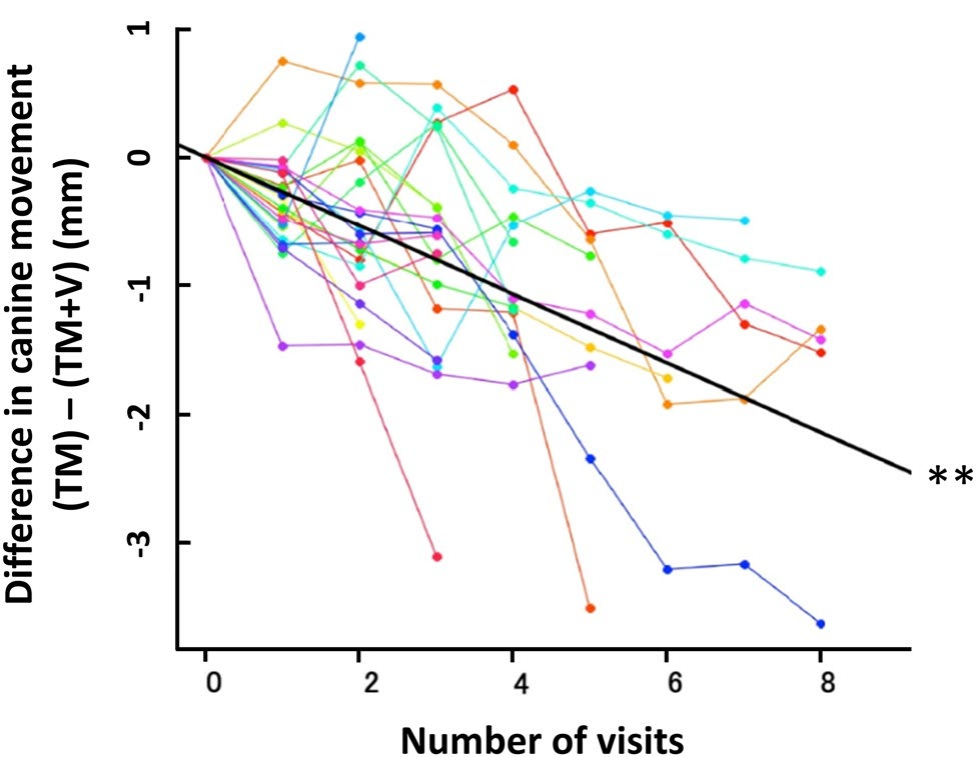
The evaluation of canine movement by LME model. The LME model of the amount of canine movement for each visit. Each colored line represents the difference in the amount of canine movement between the TM and TM + V group of subjects at each number of visits. The result of the fixed effect incorporated in each subject as a random effect is shown as a black line. The slope of the black line was -0.26, indicating a significant movement in the model. **P < 0.01.

The results using the linear mixed effect model (LME model) are shown in Fig. 3. Each color represents a subject and the black line shows the regression including the random effect. The fixed effect (slope) of the LME model was −0.26 for the amount of canine movement, indicating a significant difference in canine tooth movement between the TM group and the TM + V group (**P < 0.01).

### Reduction in the number of visits by supplementary vibration

**Figure 4:**
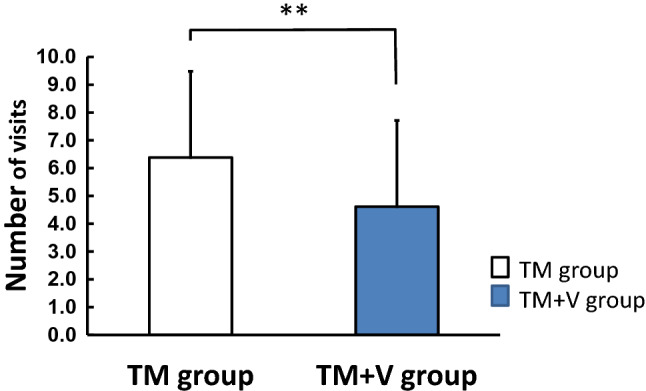
Estimated number of visits. TM + V group was estimated to have a lower number of visits than the TM group. **P < 0.01.

Figure 4 shows the estimated number of visits required to close the extraction space. The number of estimated visits for space closure was 6.38 ± 3.10 (95% CI: 5.00 to 7.76 times) in the TM group and 4.61 ± 2.15 (95% CI: 3.66 to 5.57 times) in the TM + V group (Fig. 4). The number of visits in the TM + V group was significantly less than that in the TM group (**P < 0.01). The difference between the number of visits in the TM and TM + V groups was 1.77 ± 4.65 (95% CI, 1.34 ± 2.19 times).

### No side effect of supplementary vibration in canine movement

**Figure 5:**
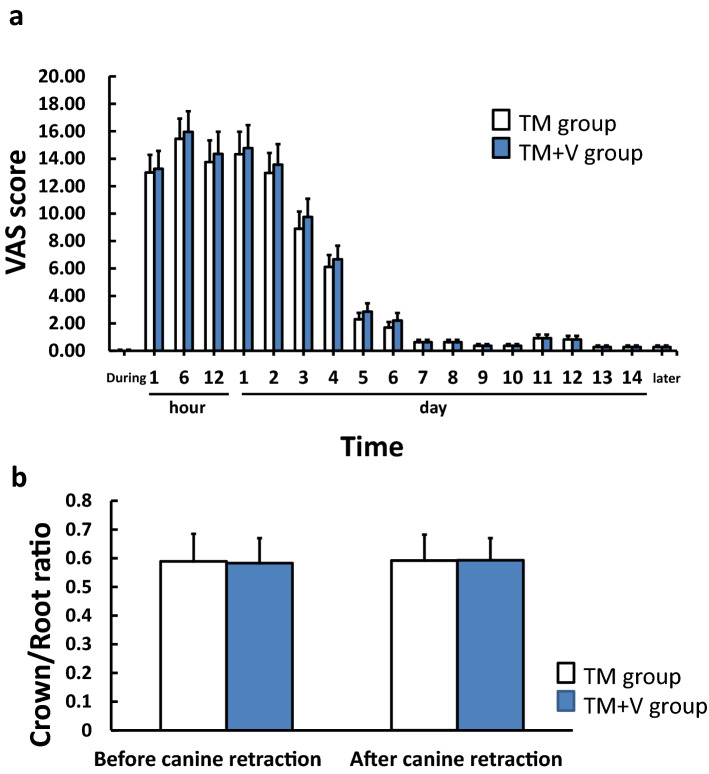
Evaluation of pain and root resorption. **(a)** VAS score. The horizontal axis indicated the period during and after the vibration, and the vertical axis indicated the VAS score (maximum value: 100). The VAS score increased from 1 h after the vibration and reached a maximum value at 6 h, after which VAS score decreased in the TM and TM + V groups. The mean VAS score did not exceed 1 mm after the 7th day. No significant difference was observed between the TM and TM + V groups at all time points. **(b)** Changes in crown root ratio before and after canine retraction.

No subjects complained of any pain during 3 min of supplementary vibration with static orthodontic force. In the evaluation of VAS scores, no significant difference was observed between the TM and TM + V groups at all time points (Fig. 5a). VAS scores of both groups peaked at 6 h (15.45 ± 14.66, 95% CI, 8.17 to 22.74; 15.95 ± 15.01, 95% CI, 8.49 to 23.42, respectively) to 24 h (14.32 ± 16.35, 95% CI, 6.19 to 22.46; 14.78 ± 16.69, 95% CI, 6.48 to 23.08, respectively); then, they gradually decreased, and returned to the baseline by 7 days. Three of the subjects did not complain of pain throughout the treatment period. No subjects reported discomfort on the questionnaire.

The dental crown root ratio at the start and the end of canine retraction is shown in Fig. 5b. The crown root ratio at the start of canine retraction was 0.59 ± 0.02 (95% CI, 0.54 to 0.63) in the TM group and 0.58 ± 0.02 (95% CI, 0.54 to 0.62) in the TM + V group (Fig. 5b). The crown root ratio at the end of canine retraction was 0.59 ± 0.02 (95%CI, 0.56 to 0.63) in the TM group and 0.59 ± 0.02 (95% CI, 0.56 to 0.63) in the TM + V group. No significant difference was observed between the two groups.

### Harms

The side effects of this study such as root resorption, pain and discomfort were not observed in supplementary vibration group compared to control groups. Furthermore, none of the subjects showed mobility and falling out of orthodontic anchor screws, or bracket detachment during the study period. In addition, no adverse events due to the multi-bracket appliance and vibration device were noted in clinical trials.

## Discussion

In the present study, the vibration characteristics and method of applying vibration to humans were determined based on the previous animal study of our group^27^. We have applied vibration of 1 gf at 58 Hz, 3 gf at 70 Hz or 50 gf at 268 Hz for 3 min every week with continuous static force during tooth movement for 21 days in rats^27^. As a result, the amount of tooth movement was significantly higher in the 3 gf at 70 Hz group than in other groups; on day 21, the amount of tooth movement was about twice as large as those of other groups. Furthermore, in order to evaluate the effect of various durations of exposure to vibration on tooth movement, the most effective vibration, 3 gf at 70 Hz, was applied weekly for 3, 6, 10 or 30 min in rats. No significant difference was observed among them during tooth movement^27^. These findings suggest that excessive force and a longer duration of vibration did not lead to an effective acceleration of orthodontic tooth movement.

In this double-blind prospective randomized controlled clinical trial, we demonstrated small intervention and great accelerating effect of supplementary vibration applied for teeth as a mechanical dynamic stimulus based on animal experiments. In rats, the duration of the remodeling cycle of alveolar bone is approximately 7 days^29^. It was reported that one cycle of human bone remodeling is two to five months, and that the bone resorption phase by osteoclasts is two to four weeks^30^. Based on these findings, for applying the vibration to humans in this study, we set a vibration force of 5.2 ± 0.5 gf at 102.2 ± 2.6 Hz and a vibration period of 3 min at monthly visits and evaluated the efficacy and safety of the acceleration of tooth movement. The vibration force used in this study was about one-fifth as great as that of AcceleDent™, and the duration of vibration application and the number of applications were less than the daily 20-min use of AcceleDent™. In this study, a smaller vibration force, shorter duration, and fewer applications were chosen to have a sufficient effect of promoting tooth movement without side effects; this was less invasive manner than the conventional vibration device AcceleDent™. Thus, the efficacy and safety of this prototype vibration device has been proven in the present study.

In this study, we focused on the canine retraction stage, which can provide a single retraction force to canine. Canines, having the longest root length and the largest root surface area other than molars, are difficult to move^31^. Therefore, if good results are obtained at the canine retraction stage, it is presumed that a similar promoting effect can be obtained on teeth at other treatment stages. In addition, a split-mouth design, which can obtain both experimental and control sides in the same subject, was performed. This design is effective for comparing orthodontic treatment effects as it reduces individual differences^32^. Furthermore, vibration was applied under double blinded conditions as neither the subjects nor the orthodontists were informed of the vibration side. Two cases with occlusal interference during canine movement were excluded. Thus, the randomized controlled trial of this study allowed us to verify the effect of accelerating tooth movement more accurately and reproducibly than previous reports.

In the present study, to precisely measure the amount of canine movement at each visit, the dentition model was converted into digital data by 3D scanner and superimposed on the reference area using analysis software. It was previously reported that the soft tissue change of third palatal rugae was stable throughout orthodontic treatment^33,34^. Recently, several researchers searched the stable region of soft tissue throughout orthodontic treatment as a superimposed area^35,36^. Jangs et al.^36^ showed a superimposed area using three orthodontic anchor screws in the palate area as reference and measured soft tissue change during orthodontic treatment. Similarly, Chen et al.^35^ used six orthodontic anchor screws as reference, showing the palatal region for superimposition, and evaluated the outcome of treatment. In the present study, we used both the region proposed by Chen et al.^35^ and occlusal surface regions of the maxillary right and left second molars. Thus, the accurate and reproducible superimposition by using these regions allowed a correct assessment of canine movement.

In clinical trials and various experiments, data may be measured repeatedly from the same subject. Of the repeated measurement data, those whose order cannot be changed over time are longitudinal data^37^. First, we measured the cumulative amount of canine movement at every visit; as a result, a significant difference between the TM group and the TM + V group was observed, indicating that supplementary vibration accelerated tooth movement more than static force alone. The measured values of the amount of tooth movement obtained by this analysis were repeated measurement data and longitudinal data, which might be affected by individual differences such as age, gender differences, bone metabolism, and oral health conditions. Therefore, as another analysis method, we applied a linear mixed effect model with individual differences as a random effect^38^. The results showed a significant difference between the TM and TM + V groups. Thus, more accelerated canine movement by supplementary vibration compared to static force alone during orthodontic tooth movement was clearly indicated.

Regarding the amount of canine movement in each coordinate axis, evaluation of the x-axis, which represents the lateral movement of the canine, and the z-axis, which represents the anteroposterior movement of the canine, showed significantly greater values in the TM + V group than in the TM group at all visits except for the third and eighth visits on the x-axis, and the six and seventh visits on the z-axis. In the evaluation of the vertical direction of the y-axis, the reciprocating movement within ± 0.5 mm was shown. Kojima et al.^39^ reported that canine movement showed simultaneously in both tipping and bodily movement by using finite element analysis in a canine retraction model. Real canine movement in the present study was similar to Kojima et al.’s model. Since supplementary vibration accelerated the canine movement, it was statistically calculated and the number of visits for canine retraction was estimated. As a result, supplementary vibration significantly reduced the number of visits required for canine retraction during orthodontic treatment.

Orthodontic tooth movement differs between subjects, and there are various individual factors among subjects such as age, gender, bone metabolism, oral environment, and periodontal condition^40–43^. Some studies have reported that vibration accelerates orthodontic tooth movement^21,22^, but others have reported that it does not^24,25^. The problem with previous research may be that the study design and assessment of tooth movement were ambiguous and unclear, and that the vibration characteristics and application method were not decided based on biological background for tooth movement. Woodhouse et al.^24^ reported that there was no significant difference in treatment between a group using Acceledent™ and a control group in the change of crowding at the leveling stage. However, in their study, the accuracy of AcceleDent™ use was questionable, because the patients applied vibration daily at home by themselves, and there was less certainty and reproducibility in the method of measurement of tooth movement. Therefore, accurate measurement of tooth movement was not possible. Furthermore, the precise effect was difficult to obtain, because the visit interval was as long as 6 weeks, which is longer than that of usual orthodontic treatment. DiBiase et al.^25^ also reported that AcceleDent™ had no effect in the space closing stage; however, there remained the problems of no description of anchorage, ambiguous retraction force, and the long visit interval of 6 weeks. These reports are still controversial. In the present study, by using this new vibration device, orthodontists applied the vibration at the monthly visit, and it is easier to obtain the accelerated tooth movement safely, certainly, and efficiently. Moreover, it eliminates the burden for patients who apply vibrations using AcceleDent™ for 20 min every day at home by themselves. Then, we clearly showed that supplementary vibration significantly accelerates the rate of canine movement and reduces the estimated number of visits for treatment.

Regarding root resorption, we investigated the changes in crown root ratio before and after the start and end of canine retraction in the TM and TM + V groups, and no significant difference was found between the two groups. Then, it was clearly demonstrated that supplemental application of vibration did not affect root resorption during the canine retraction. Kau et al.^44^ and DiBiase et al.^45^ also evaluated root resorption in the measurement of the leveling stage using AcceleDent™ and reported that there was no significant difference between the AcceleDent™ group and the control group. However, root resorption in their studies was assessed in subjects who did not show accelerated tooth movement using AcceleDent™. Therefore, the accelerated tooth movement without side effects in the present study is considered to have a high clinical significance and to be an important, previously unreported, finding.

Bergius et al.^46^ reported that the pain after orthodontic treatment increased in 4 to 24 h and had decreased to normal level 7 days later. Immediate pain corresponds to the sensation that occurs immediately after the periodontal tissue is compressed by orthodontic force, while delayed pain occurs approximately one day after orthodontic force is applied. Our results were similar to theirs, and no difference in pain or discomfort was observed between the TM group and the TM + V group during the study period, suggesting no increase in pain or discomfort associated with supplementary vibration. Animal studies have shown that applying orthodontic force to teeth causes biphasic pain: immediate and delayed pain, with contralateral and ipsilateral cross-reactivity in the obliteration and central regions^47^. Furthermore, delayed pain was more emotional than epistemic^48^. Therefore, it might be difficult to make an accurate assessment of pain and discomfort with tooth movement due to individual differences in pain history.

We recognize that the sample size in this study is relatively small, however it is considered sufficient to determine the efficacy of supplementary vibration, which is the primary endpoint of the study. The observed effect sizes are similar to other studies reported for randomized trials using AcceleDent^22,24,25,45^. Studies with larger sample sizes are needed to confirm our findings, even if the significant results obtained in this study are justified.

In conclusion, a double-blind prospective randomized controlled trial in this study demonstrated that static orthodontic force with supplementary vibration of 5.2 ± 0.5 gf at 102.2 ± 2.6 Hz for 3 min accelerated tooth movement in canine retraction and reduced the number of visits. Side effects such as root resorption and pain were not observed during the study period. The findings of this study indicated that patients' quality of life could be improved by reducing the risk of dental caries, periodontal disease and root resorption, pain and discomfort by shortening the long-term treatment period of current orthodontic treatment. In addition, vibration promotes not only tooth movement but also bone resorption, formation, and remodeling, and then, it is expected to be applied to the treatment of other bone diseases in the future.

## Methods

### Trial design

This study was a single-center, prospective randomized clinical trial. No changes occurred during the trial. The study protocol was reviewed and approved by the institutional board of Tohoku University (approval number: 23-26, registration date: April 20, 2012). This study has been registered in UMIN Clinical Trials Registry (UMIN-CTR, approval number: UMIN000013722, registration date: 15 April, 2014). The authors certify that this trial has received ethical approval from the appropriate ethical committee as described above. The informed consent was obtained from all the participants prior to the study. For participants less than age 16, the informed consent was obtained from the participants and their parent/guardians. The present study followed the world medical association declaration of Helsinki: Ethical principles for medical research involving human subjects.

### Sample size calculation

The calculation of sample size was based on the data evaluating canine movement^49^. Deguchi et al.^49^ conducted a randomized controlled trial using a split-mouth design similar to the present study to evaluate the effects of low-friction attachment during canine retraction. According to their study, a 20% increase in the amount of tooth movement due to the application of vibration was assumed, with 90% power and a 5% significance level; it was calculated that 23 subjects were needed using R statistics software (R version 3.0.3, R Foundation for Statistical Computing, Vienna, Austria).

### Participants, eligibility criteria, and settings

Subjects were patients with malocclusion after orthodontic diagnosis who visited the Orthodontic Clinic of Tohoku University Hospital from September 2012 to February 2014. A total of 341 patients were included in the study (Fig. 1). Informed consent was obtained from all subjects. Exclusion criteria were patients with (1) congenital or systemic disease; (2) skeletal facial asymmetry; (3) missing teeth; (4) serious periodontal disease; (5) temporomandibular joint disorder; (6) bruxism. All diagnoses were performed by one supervisor doctor (T.T-Y) and included 27 subjects with extracted maxillary first premolars and two retracted upper canines. The final number of subjects was 25. The split-mouth design was used to compare the amount of movement of the left and right canines of the subjects. The one side with only static force for tooth movement was classified as the non-vibration group (TM group, n = 25), and the other side with static force and supplementary vibration was classified as the TM plus vibration group (TM + V group, n = 25).

### Randomization

One analyst (A.M.) randomly assigned the left and right canines to the non-vibration (TM) group and the vibration (TM + V) group using the statistical analysis software R version 3.0.3 (R Foundation for Statistical Computing, Vienna, Austria) with sequentially numbered, opaque, sealed envelopes. The amount of tooth movement was analyzed blindly using a three-dimensional reconstructed model.

### Vibration device

**Figure 6:**
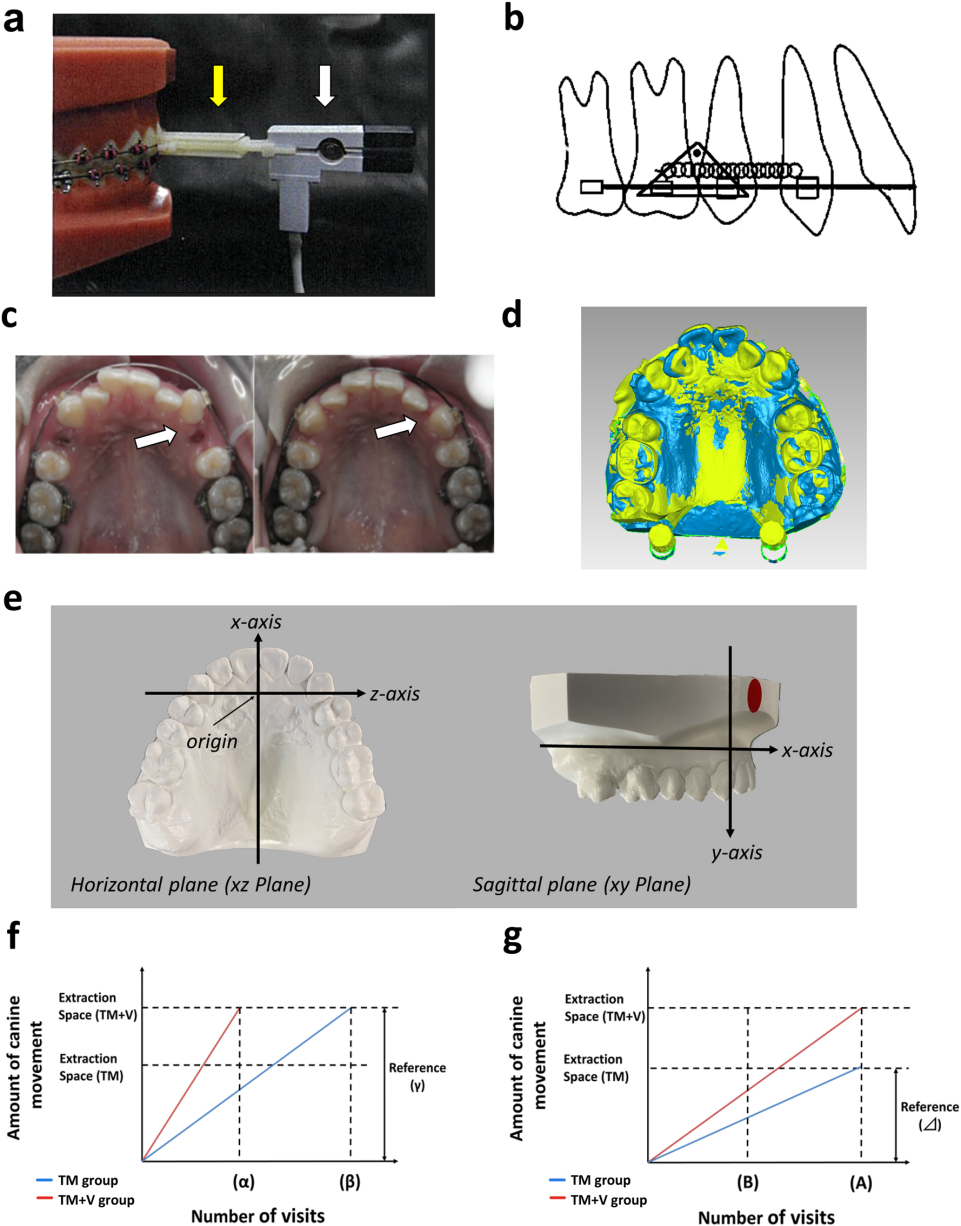
Methods of canine retraction, analysis of canine movement, and calculation of estimated number of visits. **(a)** The vibration generator and the attachment for the bracket. After the attachment (yellow arrow) was connected to the canine bracket, the vibration generator with a rotary motor (white arrow) was connected to the attachment, and vibration was applied to the canine. **(b)** Canine retraction schema. Orthodontic anchor screw, maxillary first molar and second premolar were connected using ligature wire, and canine retraction was performed with a closed-coil-spring with force of 100 gf. **(c)** Intraoral photographs during canine retraction. The left photograph is at the start of canine retraction and the right photograph is at the third visit. In this subject's case, the left canine (TM + V group) showed significantly greater movement than the right canine (TM group). **(d)** Image of superimposed model of canine retraction. A blue image indicates the start of canine retraction and the green image indicates during canine retraction. The palatal region and occlusal surfaces of the maxillary second molars were used as reference regions for superimposition. **(e)** Method of setting the coordinate axis. The x-axis was set parallel to the median palatal suture line, and the origin was the intersection of the x-axis and the incisal papillae. The occlusal plane was set to the xz plane and the plane perpendicular to the xz plane was set to the xy plane. These coordinate systems were used to disassemble the canine movement into x-, y-, and z-axes. **(f,g)** Calculation of estimated number of visits. Estimated number of visits were calculated using the average amount of canine movement for each subject. **(f)** If the extraction space of the TM + V group closed first, the extraction space (γ) of the TM + V group was used as the reference. The number of visits in which the TM group reached the distance (γ) was estimated. **(g)** If both extraction spaces are closed at the same visit, both groups finished the canine traction at time (A). In this case, the extraction space (Δ) of the TM group with the shorter moving distance was used as the reference. The number of visits (B) in which the TM + V group reached the distance (Δ) was estimated.

The vibration device used in this study consisted of a controller, vibration generator with a rotation motor, an eccentric vibration motor^27^ and the attachment for the bracket slot as shown in Fig. 6a. The force magnitude of vibration was 5.2 ± 0.5 gf and the frequency was 102.2 ± 2.6 Hz. The load applied to the tooth from the vibration generator was evaluated by using a measuring jig containing a load cell (LTS500GA, Kyowa Electronic Instruments Co., LTD, Tokyo, Japan). The vibration was applied for 3 min in the TM + V group at the monthly visit under double-blind conditions (Fig. 6a).

### Interventions

All subjects were treated under one orthodontic instructor (M.S.) at the Orthodontic Department of Tohoku University Hospital. After the upper right and left first premolars were extracted, the pre-adjusted 0.018-in. slot brackets with Roth-prescription (Crystabrace7, Dentsply Inc, Tokyo, Japan) were bonded to the bilateral canines and the second premolars, and tubes were attached to all first and second molar bands of all subjects (Double convertible tube, Dentsply Inc, Tokyo, Japan). All subjects were started leveling with 0.014-in. nickel titanium full archwire (Broad Arch NiTi Archwire, Ormco Inc, CA, USA). Average time of leveling was 5.5 ± 2.7 month (min: 2.9, max: 14.5). Orthodontic anchor screws (diameter: 1.4 mm, length: 5 to 7 mm, Absoanchor, Dentos Inc, Daegu, Korea) were implanted at the attached gingiva between the upper second premolar and the first molar as absolute anchorage.

The canine retraction was started using a 0.016 × 0.022 stainless steel preformed archwire (Broad Arch Stainless Steel Archwire, Ormco Inc, CA, USA). A NiTi closed coil spring (Sentalloy Coil Springs, Tomy International Inc, Tokyo, Japan) was attached to the bracket hook of the first molar and canine with static force of 100 gf (Fig. 6b,c). The vibration was applied to the canine in the TM + V group. Canines were not allowed to occlude the mandibular dentition, and the vibration was applied without the attachment contacted with the lips or other devices.

Subjects visited the hospital once a month for orthodontic treatment, and a maxillary dentition impression was taken using an alginate impression material (Algiace Z, Dentsply Inc, Tokyo, Japan).

### Outcomes

The primary outcome of this study was to verify the effect of vibration application by comparing the amount of canine movement and the number of visits required for canine retraction in the TM and TM + V groups.

The secondary outcome was to verify the safety of vibration application by comparing the side effects such as pain, discomfort, and root resorption in the two groups.

### Construction of three-dimensional analysis

All the plaster models were taken at every monthly visit and converted to three-dimensional models using a 3D scanner (Rexcan DS2, Solutionix, Seoul, Korea) connected to a Windows PC (Core i7 4770 K 3.9 GHz, DDR3 8 GB, GeForce GTX1070, Windows 10 Pro). The maximum reading accuracy was 0.02 mm and the reading was saved in STL format. Dental models of each subject were superimposed with 3D analysis software (Geomagic Qualify 2013, 3D systems, NC, USA) and canine movement was measured. The region of superimposition was selected areas of the central part of the palate including the third palatal rugae^35^ and the occlusal surfaces of bilateral second molars without orthodontic force being applied. All reference areas were manually selected by single operator (A.M.). The best-fit algorithm^50^ was performed using reference areas by aligning 1000 randomly selected data points, which was then refined with an alignment on 2500 data points.

As according to Cha’s method, the origin and axis setting was based on the model at the start of canine retraction^51^ (Fig. 6d). The x-, y- and z-axes were set to the mesiodistal, buccolingual and vertical directions (Fig. 6e). The point of origin was set at the intersection of the incisal papillae and the palatal suture. The occlusal plane was set as the plane connecting the midpoint of the incisal edge, and the distobuccal cusp of the maxillary bilateral first molars. The xz plane passed through the origin and was set to be parallel to the occlusal plane. The xy plane was set to pass through the origin and midpalatal suture and to be perpendicular to the xz plane. The yz plane was set to pass through the origin and was to be perpendicular to the xy and xz planes (Fig. 6e). Based on these coordinate systems, the three-dimensional canine movement was calculated using the coordinates of the canine cusp top as the measurement point. Furthermore, the amount of canine movement was divided into x-, y-, and z-axis components and evaluated. The position of the canine cusp top which has high reproducibility as the measurement point, was automatically selected by the software at the most protruding point.

### Evaluation of the amount of canine movement

The amount of canine movement in the TM and TM + V groups were measured by superimposed three-dimensional models. The amount of canine movement of each subject was measured using a minimum of two and a maximum of eight models from the start of canine retraction to a minimum of two visits and a maximum of eight visits, respectively.

All measurements of canine movement were repeated one week apart and the intra-class correlation coefficients (ICCs) using a one-way variable model were calculated to evaluate the intra-rater reliability. As a result, ICCs showed 0.97 for all measured records. Then, we used the first measured value.

### Evaluation of number of visits

The number of visits required to close tooth extraction space was estimated for each subject. Based on the moving distance of canines, when one side of the tooth extraction space was closed earlier, the number of visits was estimated at the time that the canine on the opposite side would reach the same distance (Fig. 6f). When the tooth extraction spaces of both groups were closed at the same time, the number of visits on the opposite side was estimated based on the side with less tooth extraction space at the start of canine retraction (Fig. 6g). The average value of the estimated number of visits required to move the canine for the space closure was calculated, and compared between the TM and TM + V groups.

### Evaluation of side effects

Twenty-three subjects were given a questionnaire using the visual analog scale (VAS) method^52^ for pain at the end of treatment at each visit. Pain was noted during vibration, at 1, 6, and 12 h, and on day 1, 2, 3, 4, 5, 6, 7, 8, 9, 10, 11, 12, 13, 14, and later. The average value of each numerical value of the TM and TM + V groups was calculated and evaluated. Regarding the discomfort, subjects were asked to describe in the survey form how much discomfort they felt.

To evaluate root resorption, panoramic radiographs were taken before and after canine retraction for the twenty-three subjects. There was no canine tipping in all subjects on panoramic radiographs. The crown-root ratio of maxillary canines in the TM and TM + V groups was calculated^53^. The crown length was represented by the distance between the incisal edge and the cement-enamel junction on the long axis, and the root length was represented by the distance between the cement-enamel junction and the apex. All root and crown lengths were measured by one examiner under blinded conditions. All crown-root ratios were measured again one week later without knowing which X-ray image belonged to which side of each subject. ICCs using a one-way variable model were calculated to evaluate the intra-rater reliability. As a result, ICC was 0.99. Then, we used the first measurement value.

### Blinding

Vibration was applied in a separate room by a single dentist (A.M.) who was not involved in orthodontic treatment. Vibration attachments were placed on both canines of the subjects and only the canines of the TM + V group were given vibration. The attachment of canines in the TM group were connected to a non-motorized vibration generator. The treatment doctor was not informed which canine had been vibrated. Subjects were also not informed which canine (left or right) was vibrated. All measurements using 3D models were performed by one researcher (A.M.) after blinding the model in linkable anonymizing.

### Statistical analysis

All statistical analysis was performed using R statistics software (R version 3.0.3, R Foundation for Statistical Computing, Vienna, Austria). A significant difference was defined as a P value less than 0.05. Paired t-tests were performed in the amount of canine movement between the TM and TM + V groups. In addition, a linear mixed effect model (LME model)^38^ was performed using the “lme 4^54^ ” and “ lmerTest^55^ ” packages of the R software to evaluate the canine tooth movement in the TM and TM + V groups. For all subjects, the LME model was created with the difference in the amount of canine movement between the TM and TM + V groups on the vertical axis, and the number of visits on the horizontal axis. This model had a linear relationship and the slope of the model varied with individual differences in the subject’s canine movement. Individual differences were influenced by age, gender, bone metabolism, oral status, etc. Therefore, a random effect was adapted to individual differences and the origin of the LME model was fixed in the position at the start of canine retraction.

Evaluation of number of visits and root resorption were used with paired t-test. The assessment of pain using the VAS method was evaluated using one-way analysis of variance and Tukey's method.
